# The impact of post-alignment processing procedures on whole-exome sequencing data

**DOI:** 10.1590/1678-4685-GMB-2020-0047

**Published:** 2020-11-13

**Authors:** Murilo Guimarães Borges, Helena Tadiello de Moraes, Cristiane de Souza Rocha, Iscia Lopes-Cendes

**Affiliations:** 1 Universidade Estadual de Campinas (UNICAMP), Faculdade de Ciências Médicas, Departamento de Genética Médica e Medicina Genômica, Campinas, SP, Brazil.; 2 Instituto Brasileiro de Neurociência e Neurotecnologia (BRAINN), Campinas, SP, Brazil.; 3 Universidade Estadual de Campinas (UNICAMP), Centro de Engenharia Biomédica. Campinas, SP, Brazil.

**Keywords:** Sequence alignment, quality recalibration, variant discovery, BIPMed, LatinGen

## Abstract

The use of post-alignment procedures has been suggested to prevent the identification of false-positives in massive DNA sequencing data. Insertions and deletions are most likely to be misinterpreted by variant calling algorithms. Using known genetic variants as references for post-processing pipelines can minimize mismatches. They allow reads to be correctly realigned and recalibrated, resulting in more parsimonious variant calling. In this work, we aim to investigate the impact of using different sets of common variants as references to facilitate variant calling from whole-exome sequencing data. We selected reference variants from common insertions and deletions available within the 1K Genomes project data and from databases from the Latin American Database of Genetic Variation (LatinGen). We used the Genome Analysis Toolkit to perform post-processing procedures like local realignment, quality recalibration procedures, and variant calling in whole exome samples. We identified an increased number of variants from the call set for all groups when no post-processing procedure was performed. We found that there was a higher concordance rate between variants called using 1K Genomes and LatinGen. Therefore, we believe that the increased number of rare variants identified in the analysis without realignment or quality recalibration indicated that they were likely false-positives.

## Introduction

Advances in sequencing methods have made it possible to interrogate the genome in its most basic components at an affordable price and in a timely manner (Goodwin *et al.,* 2016). A single sequencing reaction generates reads that, after processing, make it possible to compare the resulting assembly against a given reference genome (Li and Durbin, 2009; DePristo *et al.,* 2011). Based on these achievements, the rapid adoption of such techniques are being directly applied to medicine, inaugurating the new era of genome medicine (Karczewski, 2013). Nonetheless, understanding the pathogenicity of a given variant is not a straightforward task and it demands prior knowledge or biological and in-silico validation (Thusberg *et al.,* 2011). In this perspective, databases containing variants previously related to disease have an unquestionable role while linking phenotypes to genotypes (Landrum *et al.,* 2018).

The common variants from a given population are also relevant to clinical diagnosis, while evidencing that normal alterations are not necessarily linked to disease (International HapMap Consortium*,* 2010; 1000 Genomes Project Consortium*,* 2015). Many such projects have been implemented by initiatives across the globe, providing a much richer picture of human variation across regions and/or populations (Haga, 2017; Wijmenga and Zhernakova, 2018; Stark *et al.,* 2019). In Latin America, one of the first initiatives was the Brazilian Initiative on Precision Medicine (BIPMed), which expanded into additional projects in the Latin American Database of Genomic Variation (LatinGen), aiming to assist with genomic data sharing in Latin America. Currently, despite being an initiative that encompasses all of Latin America, only two databases are contributing to genetic variation from the reference population (or healthy individuals), from the Brazilian population. Thus, LatinGen materializes what many studies reinforce, the need for a better understanding of the admixture in Latin American populations, as well as other underrepresented populations, in large scale sequencing studies (Ruiz-Linares *et al.,* 2014; Petrovski and Goldstein 2016; Popejoy and Fullerton, 2016; van Rooij *et al.,* 2017). 

The use of post-alignment bioinformatics procedures has been suggested to reduce false-positive discovery rates in massive DNA sequencing data (McKenna *et al.,* 2010). Insertions and deletions are most likely to be misinterpreted by the alignment algorithms, which may produce several false single-nucleotide variants in the call-set. The use of high-quality, commonly-known variants tends to minimize such mismatching and allows reads to be correctly realigned and recalibrated. This procedure tends to increase the number of true genetic variants identified (Vo and Phan, 2018). In this work, we aim to investigate the impact of using different sets of common variants as a reference to enhance variant discovery in whole-exome sequencing data.

## Material and Methods

### Common variant sites for local realignment and quality recalibration

Targets were selected for local realignment and quality recalibration from common insertions and deletions included within the 1K Genomes Project data and common variants in reference population datasets deposited in the LatinGen Databases. 

For common insertions and deletions from the 1K Genomes Project, variants from the Broad Institute resource bundle were used and was considered the best set of known indels for optimizing local realignments (“What's in the resource bundle and how can I get it?” 2020). This data was comprised of 3,989,738 indels from 1K Genomes Project Phase I and an additional 831,742 gold-standard, double-hit indels, which totaled 4,570,615 unique entries.

Data from the Latin-American Database of Genomic Variation (LatinGen) was composed of three databases. Two were from the Brazilian Initiative on Precision Medicine (BIPMed-Array-db and BIPMed-WES-db) (Secolin *et al.,* 2019; BIPMed genomic databases, 2020) and one was from the Online Archive of Brazilian Mutations (AbraOM) (Naslavsky *et al.,* 2017).

The Brazilian Initiative on Precision Medicine (BIPMed) was composed of two databases, which contained pooled variant information from subjects from the Brazilian reference population collected in Campinas, Brazil (BIPMed-WES-db). Whole-exome sequencing (WES) experiments were composed of 258 subjects and a BIPMed-Array-db containing data that were derived from microarray-based experiments involving 264 individuals (Affymetrix GenomeWide SNP 6.0 array). The BIPMed-WES-db was composed of 851,109 variants. Of these, 1282 variants (0.15%) were selected with a minor allele frequency (MAF) > 0.5, quality greater than 30, a depth of coverage higher than 10 fold, and with 90% or more of all possible alleles covered. The BIPMed-Array-db contained 906,600 variants, of which we selected 447618 (49.4%). 

The Online Archive of Brazilian Mutations (AbraOM) repository contained WES variants from 609 elderly individuals from the city of Sao Paulo, Brazil and included a total of 2,382,574 variants. Of these, we selected 1574 (0.07%) high-quality insertions or deletions that were present in more than 50% of the alleles (MAF > 0.5) examined. As quality filters, variants with substantial evidence of being true-positives were selected, flagged as “very strong” probability of being true and PASS, and detailed in the project website (Naslavsky *et al.,* 2017). By merging the three databases, 450,474 unique entries were identified.

Variants from the Online Archive of Brazilian Mutations (AbraOM) are available through the projects’ website. Variants from BIPMed are available under a Research Data Use Agreement. The datasets analyzed from the 1000 Genomes Project are available through the projects’ repository. This study was approved by the Research Ethics Committee of the University of Campinas (UNICAMP), CAAE #12112913.3.0000.5404, and written informed consent was obtained from each participant of the BIPMed datasets.

### Whole exome data selected for variant calling

WES data from 122 unrelated Brazilian individuals who were initially recruited for the investigation of genetic epilepsies (Group BR) were selected. In this dataset, WES was performed following the recommended protocol provided by the manufacturer of the SureSelectXT Human All Exons V6 kit (Agilent Technologies). All 122 samples were quantified, qualified, diluted, and sent for library preparation and sequencing using an Illumina Hiseq 2500 platform in which a paired-end sequencing reaction with 101 x 2 cycles (3,605,583,845 sequences) was employed. All data generated from the BR group was disidentified, and therefore, treated as non-personal information (Lei Geral de Proteção de Dados Pessoais (LGPD), 2018, The European Parliament and of the Council, 2016). In addition, we also used 15 unrelated WES datasets, which were randomly selected from phase three of the 1K Genomes Project, which was sequenced at the same sequencing center (BGI, Beijing Genomics Institute). We chose five samples from each of the two South American populations represented in the 1K Genomes Project database, which included both Colombians from Medellin (Group CLM: HG01119, HG01142, HG01281, HG01363, HG01431, 732422573 sequences) and, Peruvians from Lima (Group PEL: HG02102, HG02150, HG02253, HG02262, HG02312, 540035325 sequences). An additional 5 European samples were selected from British populations in England and Scotland (Group GBR: HG00110, HG00121, HG00139, HG00254, HG00259, 634,824,750 sequences). We chose the number of samples studied considering the limitation of storage and processing capabilities, so that a balance in the number of sequenced bases was achieved. 

### Alignment, realignment, variant recalibration, and variant calling

Sequences were extracted from binary alignment files (BAM) provided by the 1K Genomes Project for CLM, PEL, and GBR groups, as well as those we generated for Group BR. A paired-sequence alignment of the four groups was performed using BWA-MEM (version 0.7.12) against the Human Reference Genome GRCh38 with default parameters (Li and Durbin, 2009; Schneider *et al.,* 2017). We used Picard Tools (version 2.5.0) for marking duplicates and indexing (Picard web page, 2020). The variant calling process was carried out using three different strategies: (i) without performing realignment and quality recalibration steps against the realignment, using targets selected from (ii) the 1K Genomes and (iii) LatinGen. The Genome Analysis Toolkit (version 3.6-0) was used to perform the local realignment, quality base recalibration, and variant calling steps (GATK modules: RealignerTargetCreator, IndelRealigner, BaseRecalibrator, PrintReads, and HaplotypeCaller) for both the LatinGen and 1K Genomes Project targets. The HaplotypeCaller module was used for the call-set without realignment or quality recalibration. Variants with quality values lower than 30 and coverage lower than 10 times were filtered out. To annotate variants, the Variant Effect Predictor (VEP) program (version 94) was used to determine global minor allele frequencies (GMAF) of the 1K Genomes dataset with just one selected consequence per variant (McLaren *et al.,* 2016).

**Figure 1 -:**
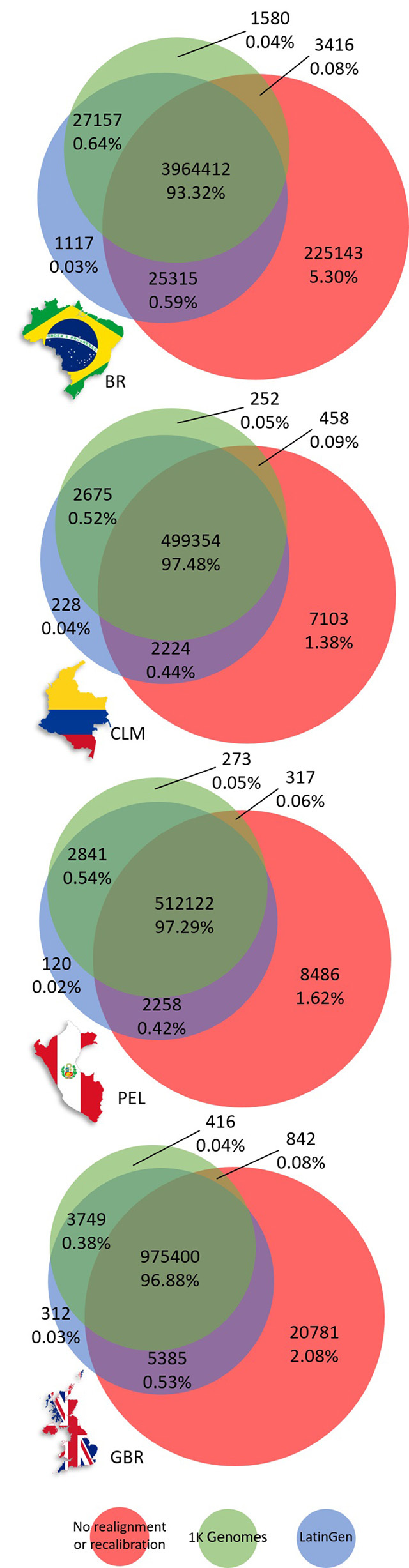
Venn diagrams for each variant calling approach: Local realignment and quality recalibration was performed using known, high-quality sites from the 1K Genomes Project and LatinGen data, which were applied to four populations: Brazilians (BR), Colombians (CLM), Peruvians (PEL), and British (GBR). The intersections of variants called were compared using three different realignment and recalibration methodologies, which included (i) variants called in the absence of realignment and recalibration steps, and those obtained using local realignment and quality recalibration using common insertions and deletions from (ii) the 1K Genomes Project, and (iii) the common variants from LatinGen. A comparison of the call-sets revealed a high degree of concordance. The increased number of variants identified exclusively when data was not subjected to realignment or recalibration may indicate that these variants are false-positives. The high degree of intersection between the call-sets that were realigned and recalibrated using 1K Genomes and LatinGen highlights the benefit of using this methodology. The implementation of local realignment and quality recalibration with an admixed population from LatinGen did not affect the four population groups considered.

The level of concordance among the variants called was established using the three following realignment and recalibration methodologies: (i) called in the absence of realignment and recalibration steps; ii) obtained from local realignment and quality recalibration steps using the 1K Genomes Project data; and (iii) obtained from the local realignment and quality recalibration of common variants from LatinGen. To assess concordance, the data is presented using Venn diagrams, with absolute and percentage values shown (Figure 1). 

Three sets of variants were evaluated in a more detailed manner. The group that was not subjected to realignment or recalibration contained uniquely called variants when no realignment or recalibration occurred. Group 1K Genomes consisted of variants identified from the realignment and recalibration with the 1K Genomes targets that were absent when using variants from LatinGen. Finally, Group LatinGen contained variants identified from realignment and recalibration using LatinGen that were absent when using variants from 1K Genomes targets. We accessed whether variants were novel by searching for their presence or absence within the dbSNP database and GMAF and through variant annotation with VEP. 

To test whether there were differences between the distributions of variants identified using the three protocols proposed for our four different population groups, the paired Mann-Whitney U Test without continuity correction was applied. To test if there were no differences between the allele frequencies of independent groups, the unpaired Mann-Whitney U test with the continuity correction was used. A p-value < 0.05 was considered statistically significant. All analyses were conducted using R statistical software (version 3.4.3) (R Core Team, 2014). VennDiagram (1.6.0), ggplot2 (2.2.1.9000), and plotly (4.7.1) packages were used to create the figures. 

## Results

Three different pre-processing protocols were applied to WES data. First, variants were called in the absence of realignment and recalibration steps. Second, variants were called using local realignment and quality recalibration steps that employed common reference variants, insertions, and deletions deposited in the 1K Genomes Project database. Third, variants were called using local realignment and quality recalibration steps that employed common reference variants, insertions, and deletions deposited in the LatinGen database. Each of the protocols described above was applied to the four populations assessed, which included BR, PEL, CLM, and GBR. 

Overall, the results showed concordance levels that ranged from 93.32% to 97.48% among the three different variant-calling protocols and population groups. When data were discordant, we found that 1.38% to 5.30% of variants were identified exclusively when no realignment or recalibration was performed. Furthermore, when comparing all the variants within our call-set by using both reference databases, 0.04% to 0.05% of the variants were exclusively identified using data from 1K Genome Project and 0.02% to 0.04% of the variants were identified solely using LatinGen (Figure 1). Of the variants called using LatinGen as a reference, 0.38% to 0.64% were also identified using the 1K Genome Project as a reference, and were not detected when post-processing procedures were omitted. Furthermore, 0.42% to 0.59% of the variants called using LatinGen as a reference were also identified when post-processing procedures were omitted but were not identified when the 1K Genome Project was used as a reference. Figure 1 shows a summary of these findings using Venn diagrams to depict the results obtained using four different populations. There were no differences observed in the distribution of variants called using any of the three protocols for the four populations (p-values > 0.4). 

**Figure 2 -:**
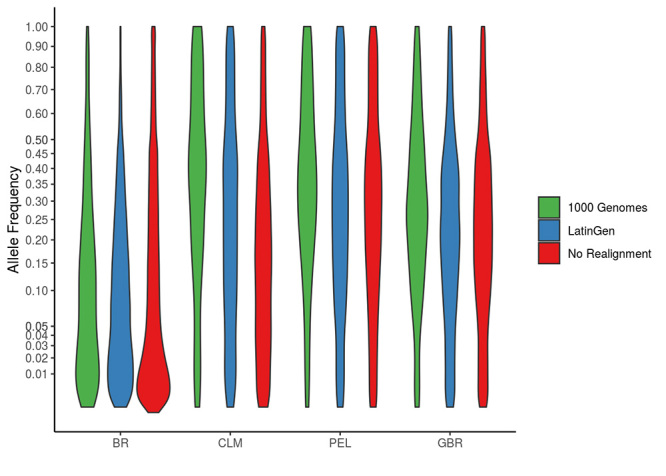
Global minor allele frequency (GMAF) distributions. A comparison of GMAF distributions within subsets of variants produced by performing no realignment or recalibration steps and those identified after realignment and recalibration using 1K Genomes targets and LatinGen for four population groups: Brazilians (BR), Colombians (CLM), Peruvians (PEL), and British (GBR). The GMAF distribution within Group BR is lower than the other populations, indicating a small level of representation of the population in genomic databases. Table 1 shows mean values with standard deviations for each variant subset.

A more detailed analysis of the unique variants called using the three different protocols is included in Table 1. In general, most of the variants identified were annotated as known (an average of 90.54%). Notably, there were lesser known to novel variant ratios in all population groups when LatinGen variants were used as the reference for post-processing procedures, which ranged from 3.79% to 6.18%. In addition, a lower number of novel variants were identified within the BR population and those known variants had lower GMAF values (mean of 0.16) than other populations (mean of 0.32; p-value<0.05 with pair-wise comparisons). Figure 2 depicts these GMAF distributions using violin plots.

**Table 1 -:** Variant description and annotation: A detailed description of the variants uniquely called when no realignment or recalibration occurred, those from the realignment and recalibration with the 1K Genomes targets, but absent when using the variants of LatinGen and, finally those from the realignment and recalibration with LatinGen, but absent when using variants from the 1K Genomes targets are summarized. WES data from 122 unrelated Brazilian individuals who were initially recruited for the investigation of genetic epilepsies (Group BR) was used. Five samples were chosen from each of the two South American populations represented in the 1K Genomes Project database, which included both Colombians from Medellin, Colombia (Group CLM) and, Peruvians from Lima, Peru (Group PEL). Five European samples were selected from British populations in England and Scotland (Group GBR). General statistics, variant classes, and coding consequences are described.

	Group BR			Group CLM			Group PEL			Group GBR		
Groups	No Realignment or Recalibration	1K Genomes	LatinGen	No Realignment or Recalibration	1K Genomes	LatinGen	No Realignment or Recalibration	1K Genomes	LatinGen	No Realignment or Recalibration	1K Genomes	LatinGen
Total Variants	225143	4996	26432	7103	710	2452	8486	590	2378	20781	1258	5697
Novel	31987	908	1633	478	47	93	780	112	195	1085	150	244
	14.21%	18.17%	6.18%	6.73%	6.62%	3.79%	9.19%	18.98%	8.20%	5.22%	11.92%	4.28%
Existing	193156	4088	24799	6625	663	2359	7706	478	2183	19696	1108	5453
	85.79%	81.83%	93.82%	93.27%	93.38%	96.21%	90.81%	81.02%	91.80%	94.78%	88.08%	95.72%
Annotated with the 1K Genomes GMAF	138131	2595	21167	4172	299	1593	5001	235	1550	14770	619	4443
	61.35%	51.94%	80.08%	58.74%	42.11%	64.97%	58.93%	39.83%	65.18%	71.07%	49.21%	77.99%
Average GMAF	0.1733	0.1665	0.1367	0.2315	0.4138	0.3143	0.3323	0.4014	0.3327	0.2706	0.3253	0.2636
GMAF standard deviation	0.2321	0.2106	0.1644	0.2403	0.2960	0.2833	0.2734	0.2791	0.2797	0.2352	0.2450	0.2276
Overlapped Genes	28081	2535	10256	4751	503	1740	4941	342	1546	9130	782	3266
Overlapped Transcripts	30383	2574	10742	4780	503	1749	5020	345	1556	9461	783	3368
Overlapped Reg. Features	6432	201	1433	121	16	80	235	11	65	988	42	296
Coding Consequences												
Missense variant	54%	42%	63%	60%	67%	42%	59%	50%	49%	62%	69%	59%
Synonymous variant	27%	38%	31%	23%	33%	52%	32%	22%	32%	33%	23%	39%
In-frame deletion	2%	4%	2%	7%	0%	6%	6%	6%	8%	1%	8%	0%
In-frame insertion	3%	4%	1%	1%	0%	0%	1%	0%	0%	0%	0%	0%
Frameshift variant	8%	6%	1%	6%	0%	0%	2%	22%	8%	2%	0%	1%
Stop gained	2%	2%	2%	3%	0%	0%	0%	0%	3%	2%	0%	0%
Others	4%	4%	0%	0%	0%	0%	0%	0%	0%	0%	0%	1%

## Discussion

Sequence aligners usually consider sequences individually as they are assembled to a reference genome (Li and Durbin, 2009; Pirooznia *et al.,* 2014). By finding a broader positional context, the local realignment tends to minimize mismatches, which produces a better consensus for variant calling (Indelrealigner: Perform local realignment of reads around indels, 2020). The purpose of quality recalibration is to assign a realistic probability to each sequenced base using a statistical model that considers the variant itself and a series of contextual parameters that support the variant-calling process (Variantrecalibrator: Build a recalibration model to score variant quality for filtering purposes, 2020). Our results revealed that an increased number of exclusive variants were identified in the call-set when no alignment or quality recalibration was performed for all the populations studied (Figure 1). Many of these variants could be false-positives due to alignment artifacts (Gézsi *et al.,* 2015; McCormick *et al.,* 2015; Hwang *et al.,* 2016). Indeed, to decrease these false-positives, there is high demand for more precise sequencing methods providing longer reads and better aligners with appropriated post-alignment processing methodologies and an appropriated coverage (Fuentes Fajardo *et al.,* 2012; Vo and Phan, 2018).

Our results show an increased number of unique variants identified when using recalibration and realignment targets from the 1K Genomes group, which could indicate an imbalanced ratio of false-positive variants when using this dataset in comparison to the targets from LatinGen. However, since there were fewer variants in LatinGen, these targets (450,474) may be less sensitive to the identification of true variants than 1K Genomes (4,570,651 targets) (Table 1), a validation step would be necessary to confirm this hypothesis. Ensuring true-positive genetic variants and establishing an association with a disease is not a straightforward task, especially for low-frequency variants (Sham and Purcell, 2014; Jew and Sul, 2019; Ross, 2020). In a call set, most of the variants tend to be benign, as unrelated samples possess normal genetic variation (Sherry, 2001; Li *et al.,* 2010). However, low-frequency variants could also have been caused by the variant-calling algorithm (Hwang *et al.,* 2016; Bomba *et al.,* 2017). 

Furthermore, our results indicate that the use of post-alignment steps may be advantageous for variant-calling and variant discovery, which are independent of the reference databases used for these procedures. Moreover, we observed a high level of concordance between variants called using the two methods for all four populations analyzed (Figure 1). Recent population growth and weak selection can increase the number of previously unknown and population-specific variants identified (Tennessen *et al.,* 2012). However, many admixed populations, such as Latin American populations, have an incipient presence in most of the public genetic and genomic databases (Genovese *et al.,* 2013; Sudmant *et al.,* 2015). Thus, we used a Latin American reference database (LatinGen) to investigate whether the use of ethnically matched common variants for post-processing alignment and quality recalibration would improve variant-calling. We found that there was no significant difference between the number of variants called using LatinGen and those identified using the 1K Genomes databases (p-values > 0.4). However, based on our findings we suggest that there is a potential benefit in the use of data from the 1K Genomes databases in combination with the ethnically matched and admixed population represented in LatinGen. This approach would take advantage of an increased sample size as well as increased genetic variability that occurs in admixed populations.

It is important to note that our study has limitations, since the number of variants included in the 1K Genomes Project was substantially larger than the number included in LatinGen datasets. However, we identified variants (1782; 0.03%) that were only called using exomes analyzed when LatinGen was used for local realignment and recalibration. Even though a biological validation of our findings, using a second technique such as Sanger sequencing, would be advisable, our results are robust. Thus, our findings should be viewed as an indication that using additional databases should be considered in the identification of genetic variants of interest when performing post-alignment steps in the analyses of massive DNA sequencing data.

In conclusion, we present and discuss the impact of using high-quality, common variants from control individuals of diverse ethnic origin on variant discovery from whole-exome sequencing data. Our results indicate that these common variants played an important role within the post-processing analyses that were applied to samples of both populations with closely and distantly related genetic backgrounds, and likely minimized the identification of false-positives while enhancing the discovery of novel variants. 
